# Kaolin Reduces ABA Biosynthesis through the Inhibition of Neoxanthin Synthesis in Grapevines under Water Deficit

**DOI:** 10.3390/ijms21144950

**Published:** 2020-07-13

**Authors:** Tommaso Frioni, Sergio Tombesi, Paolo Sabbatini, Cecilia Squeri, Nieves Lavado Rodas, Alberto Palliotti, Stefano Poni

**Affiliations:** 1 Department of Sustainable Crop Production, Università Cattolica del Sacro Cuore, Via Emilia Parmense 84, 29122 Piacenza, Italy; tommaso.frioni@unicatt.it (T.F.); cecilia.squeri@unicatt.it (C.S.); stefano.poni@unicatt.it (S.P.); 2 Department of Horticulture, Michigan State University, 1066 Bogue Street, East Lansing, MI 48824, USA; sabbatin@msu.edu; 3 CICYTEX (Junta de Extremadura), Finca La Orden, Ctra. A-V, km 372, Guadajira, 06187 Badajoz, Spain; nieves.lavado@juntaex.es; 4 Department of Agricultural, Food and Environmental Sciences, Università degli Studi di Perugia, Borgo XX Giugno 74, 06121 Perugia, Italy; alberto.palliotti@unipg.it

**Keywords:** particle film technology, xanthophylls, VAZ cycle, drought, *Vitis vinifera* L., abscisic acid

## Abstract

In many viticulture regions, multiple summer stresses are occurring with increased frequency and severity because of warming trends. Kaolin-based particle film technology is a technique that can mitigate the negative effects of intense and/or prolonged drought on grapevine physiology. Although a primary mechanism of action of kaolin is the increase of radiation reflection, some indirect effects are the protection of canopy functionality and faster stress recovery by abscisic acid (ABA) regulation. The physiological mechanism underlying the kaolin regulation of canopy functionality under water deficit is still poorly understood. In a dry-down experiment carried out on grapevines, at the peak of stress and when control vines zeroed whole-canopy net CO_2_ exchange rates/leaf area (NCER/LA), kaolin-treated vines maintained positive NCER/LA (~2 µmol m^−2^ s^−1^) and canopy transpiration (E) (0.57 µmol m^−2^ s^−1^). Kaolin-coated leaves had a higher violaxanthin (Vx) + antheraxanthin (Ax) + zeaxanthin (Zx) pool and a significantly lower neoxanthin (Nx) content (VAZ) when water deficit became severe. At the peak of water shortage, leaf ABA suddenly increased by 4-fold in control vines, whereas in kaolin-coated leaves the variation of ABA content was limited. Overall, kaolin prevented the biosynthesis of ABA by avoiding the deviation of the VAZ epoxidation/de-epoxidation cycle into the ABA precursor (i.e., Nx) biosynthetic direction. The preservation of the active VAZ cycle and transpiration led to an improved dissipation of exceeding electrons, explaining the higher resilience of canopy functionality expressed by canopies sprayed by kaolin. These results point out the interaction of kaolin with the regulation of the VAZ cycle and the active mechanism of stomatal conductance regulation.

## 1. Introduction

Global warming is rapidly changing worldwide agriculture. Growers are facing general warming trends that intensify extreme events and compromise yield and fruit quality [1,2,3]. Viticulture is one of the most relevant crops in warm and temperate regions and, in Mediterranean wine districts, multiple summer stresses (i.e., the concurrence of prolonged drought, high air temperature and excessive light radiation) are the main causes of vineyard impairments related to climate change [3,4]. The first consequence of multiple summer stresses is the reduction of carbon assimilation and transpiration (E), resulting in the loss of yield and fruit quality, according to the severity and duration of limiting conditions [4,5,6].

Kaolin (aluminium silicate) is a natural clay used for mitigating the negative impact of extreme temperatures on leaves and fruits [7,8,9]. Under non-limiting conditions, mild or no effects on assimilation and transpiration have been reported [10,11,12,13,14,15]. Under water shortage, kaolin is able to maintain better assimilation and transpiration rates, to avoid photosystem II efficiency loss and to prevent leaf photoinhibition and abscission [9,12,13,14,15]. Its mechanism of action is primarily related to the increase of light reflection that reduces the radiation absorbed by the leaf [7,8,16].

Leaf transpiration is controlled by stomata, which under water deficit are subjected to active and passive regulation. Passive regulation relies on hydraulic mechanisms mediated by environmental conditions and xylem water potential [17,18,19], whereas active mechanisms consist of biochemical signalling of abscisic acid (ABA), the accumulation of which affects stomatal closure in leaves [20,21,22].

ABA is biosynthesised in roots and leaves [23], although leaf biosynthesis seems the predominant one and the one responsible for the accumulation of ABA in leaves [24]. ABA biosynthesis proceeds from the degradation of carotenoids involved in the xanthophylls cycle (VAZ cycle) [23]. During a warm day, violaxanthin (Vx) is de-epoxidated to zeaxanthin (Zx) via the formation of antheraxanthin (Ax) [25]. The activity of Vx de-epoxide is regulated by chloroplast stroma acidification caused by the accumulation of electrons not used for the biochemical reactions of photosynthesis. Thus, the reduction of photosynthetic activity triggers the de-epoxidation of Vx, contributing to the active energy dissipation even when thermoregulation through transpiration is missing or reduced [25]. Zx is then epoxidated by the Zx epoxidase enzyme (ZEP) during a dark period, being ZEP inhibited by light [26]. On the other hand, Zx is the precursor of neoxanthin (Nx), which is formed by the activity of Nx synthase [27]. Nx is cleaved to xanthoxin and ABA-aldehyde, the reaction of which is catalysed by the 9-cis-epoxycarotenoid dioxygenase enzyme (NCED) [23]. ABA biosynthesis is induced by leaf dehydration and by the decline of cell volume [22,24]. In the ABA biosynthetic pathway, *NCED* is the gene involved in the promotion of leaf dehydration due to the increase of air-to-leaf vapor pressure deficit (VPD) [28].

Recently, in mature grapevines grown in the field, Dinis et al. [29] showed a kaolin-induced reduction of leaf ABA associated with better leaf stomatal conductance than untreated vines at leaf water potential lower than -1 MPa. Brito et al. [16] reported similar responses in olive trees.

However, the causes and the consequences of the kaolin-induced modulation of ABA biosynthesis were never investigated and the dynamic interactions between kaolin and leaf Vx, Ax, Zx and Nx under progressive water deficit are currently unknown. Since ABA biosynthesis is connected to the VAZ cycle, our hypothesis was that a lower leaf ABA in kaolin-coated leaves could be associated with a different tuning of the de-epoxidation/epoxidation state under water deficit. Therefore, the aim of the present work was to determine:

1. if Kaolin had an effect on ABA biosynthetic pathway,
2. if the eventual difference in ABA accumulation was related to possible bottlenecks on the carotenoid biosynthetic pathway that leads to ABA biosynthesis in leaves.

## 2. Results

### 2.1. Leaf Physical Properties and Vine Physiology

Kaolin coating increased leaf light reflection, increasing from 10% of the total incident radiation recorded in control vines to the 15% recorded in sprayed leaves (Table 1). Conversely, leaf-transmitted light was reduced to 6.9% of total photosynthetically active radiation (PAR) in kaolin-treated leaves vs. 8.0% found in control vines.

**Figure 1:**
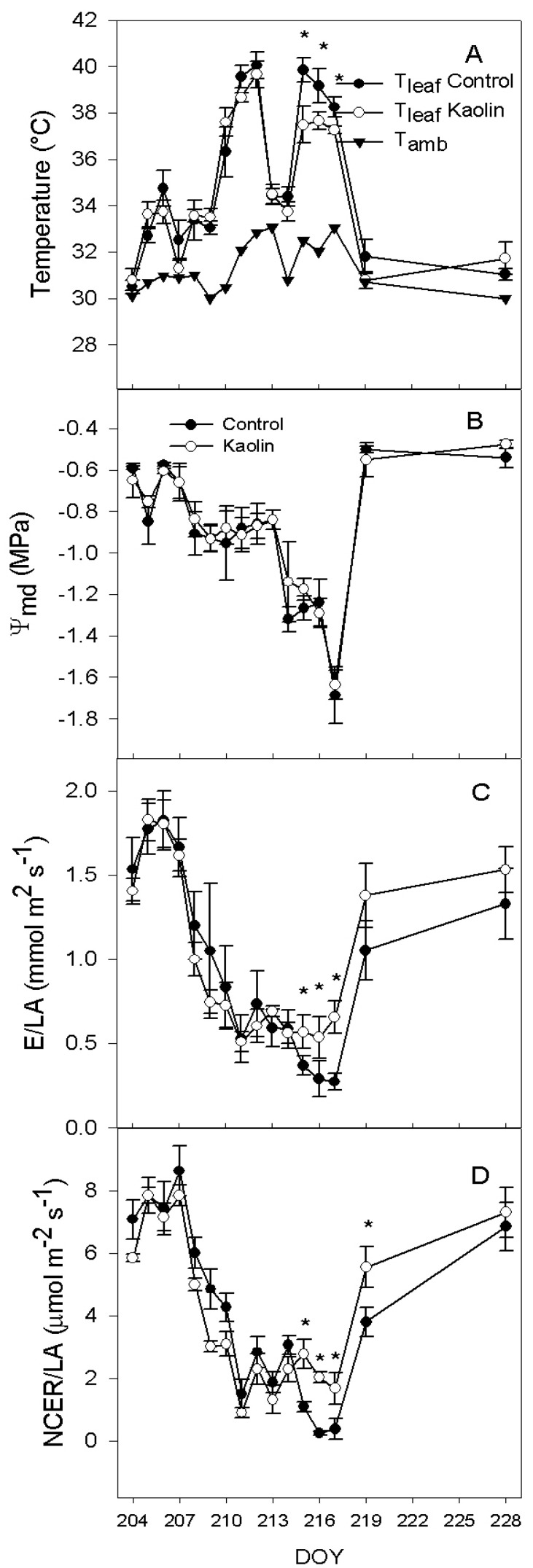
(**A**)Trends for air (T_amb_) and leaf (T_leaf_) temperature; (**B**)midday stem water potential (Ψ_MD_); (**C**) whole-canopy transpiration (E/LA) and (**D**) specific whole-canopy net CO_2_ exchange rate/leaf area (NCER/LA), according to a progressive water shortage (DOY 209–217) and subsequent re-watering (at DOY 218), in vines subjected to the kaolin treatment and in controls. Bars represents standard error (SE), *n* = 3. Asterisks indicate dates within which differences among treatment were significant (*p* < 0.05). DOY: day of the year.

Leaf temperature (T_leaf_)was similar in the two treatments except at Day Of the Year (DOY) 215 to 216 and 217 (the 3 last days of water deficit) when kaolin-coated leaves were 2.4 °C cooler than control leaves (Figure 1A). There were no differences upon re-watering (after DOY 217). Kaolin did not affect stem water potential at midday (Ψ_md)_, which decreased in both treatments from initial −0.6 MPa to −1.7 MPa on DOY 217 following the same pattern (Figure 1B). After re-watering, Ψ_md_ was restored to pre-stress value ranges. In both treatments, transpiration rate/leaf area (E/LA) decreased significantly from DOY 208 (Figure 1C). No differences between treatments were found until DOY 215, when control vines had significantly lower E/LA than kaolin vines, reaching on DOY 217 a value of 0.37 and 0.57 mmol m^−2^ s^−1^, respectively. After re-watering, no differences were found again between treatments. Net CO_2_ exchange rates/leaf area (NCER/LA) (Figure 1D) followed a similar pattern—kaolin vines maintained higher assimilation rates from DOY 215 to DOY 217 (~2 µmol m^−2^ s^−1^), whereas control vines zeroed their NCER/LA. Upon re-watering (DOY 219), kaolin exhibited higher NCER/LA than control (+46%), but this effect vanished at DOY 228, 10 days after the restoration of full water supply.

**Figure 2:**
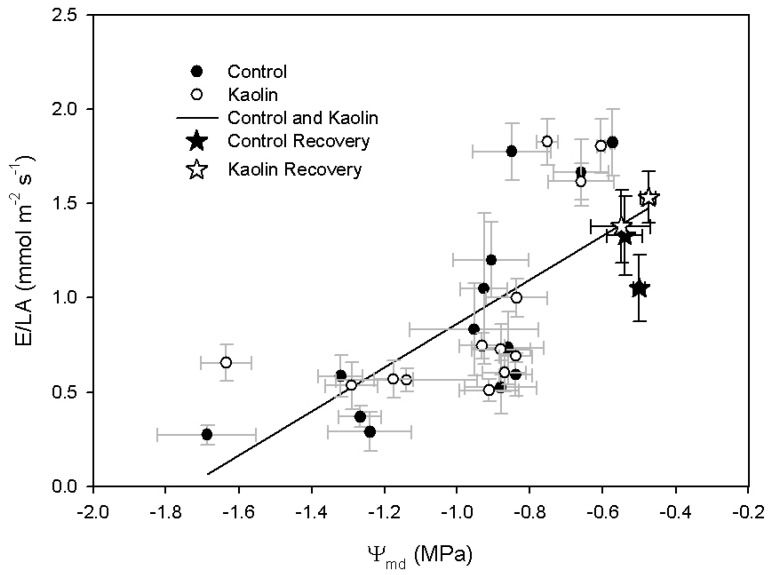
Correlation between whole-canopy transpiration rate/leaf area (E/LA) and midday stem water potential (Ψ_MD_).

No difference between treatments was found in the response of E/LA to varying Ψ_md_ after kaolin coating (Figure 2). Independently of the treatment, E/LA was positively correlated to Ψ_md_ (*y* = 1.17*x* + 2.03, *R*^2^ = 0.52, *p* < 0.05).

### 2.2. Xanthophylls and ABA Concentration

**Figure 3:**
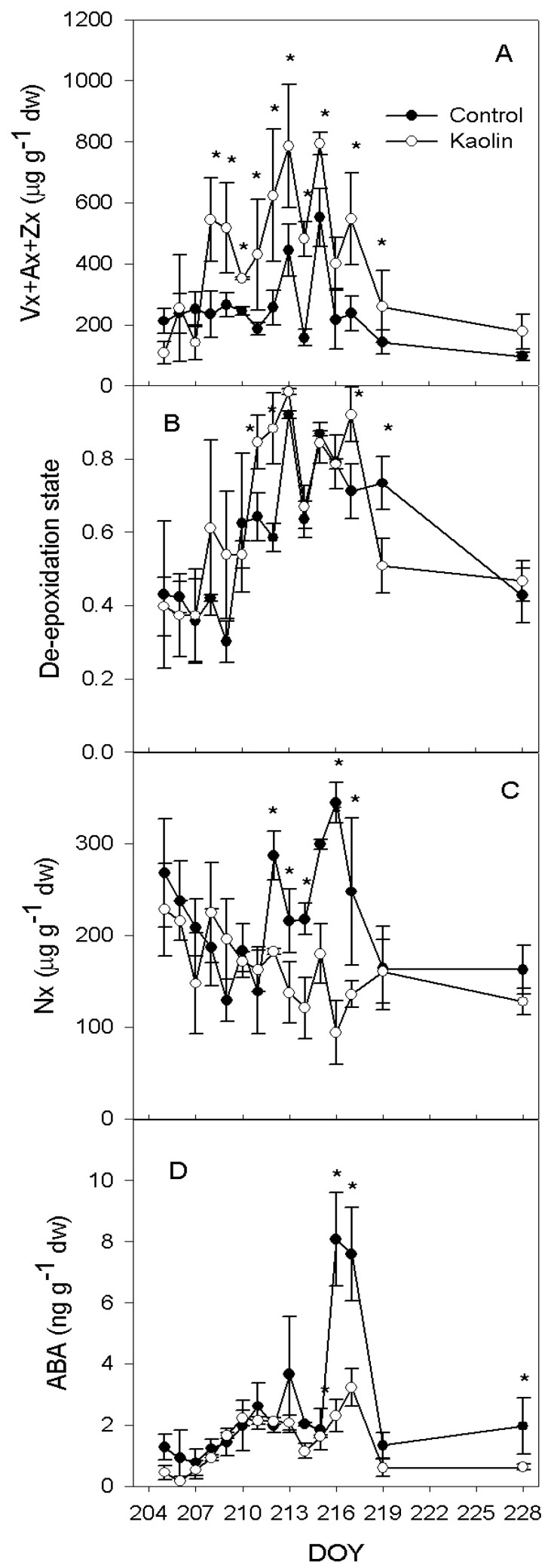
(**A**) Trends for leaf violaxanthin (Vx) + antheraxanthin (Ax) + zeaxanthin (Zx) content, (**B**) de-epoxidation state, (**C**) neoxanthin (Nx) content and (**D**) abscisic acid (ABA) concentration, according to a progressive water shortage (DOY 209–217) and subsequent re-watering (at DOY 218), in vines subjected to the kaolin treatment and in controls. Bars represents standard error, *n* = 3. Asterisks indicate dates within which differences among treatment were significant (*p* < 0.05). DOY: day of the year.

In kaolin leaves, total xanthophylls (Vx + Ax + Zx) concentration was significantly higher than control leaves from DOY 208 to DOY 219, the first day after re-watering. In this time span, the difference between treatments ranged between 107 and 367 µg g dw^−1^ (Figure 3A). Ten days after re-watering, these differences between kaolin and control disappeared. The de-epoxidation state set at about 0.4 in both treatments between DOY 205 and 207 (Figure 3B). The proportion of de-epoxidated xanthophylls was consistently higher in kaolin vs. control at DOY 211, 212 and 214, while at DOY 216 de-epoxidated xanthophylls were consistently higher in control than in the kaolin treatment.

Leaf Nx concentration was similar between treatments during the first part of the experiment (Figure 3C). However, starting from DOY 212, there was a clear course indicating higher Nx in control leaves. The difference between kaolin and control peaked on DOY 216, when Nx in control leaves was more than 3-fold that in kaolin leaves. Leaf ABA ranged between 0.5 and 2 ng g dw^−1^ in both treatments until DOY 215 (Figure 3D). On DOY 216 and 217, leaf ABA in control vines suddenly peaked up to 8.1 ng g dw^−1^, whereas in kaolin vines leaf ABA mildly increased up to 3.2 ng g dw^−1^. Right after re-watering, no difference between treatments was found; however, 10 days after the restoration of full water supply, control leaves showed again a significantly higher leaf ABA concentration (1.3 µg g dw^−1^).

**Figure 4:**
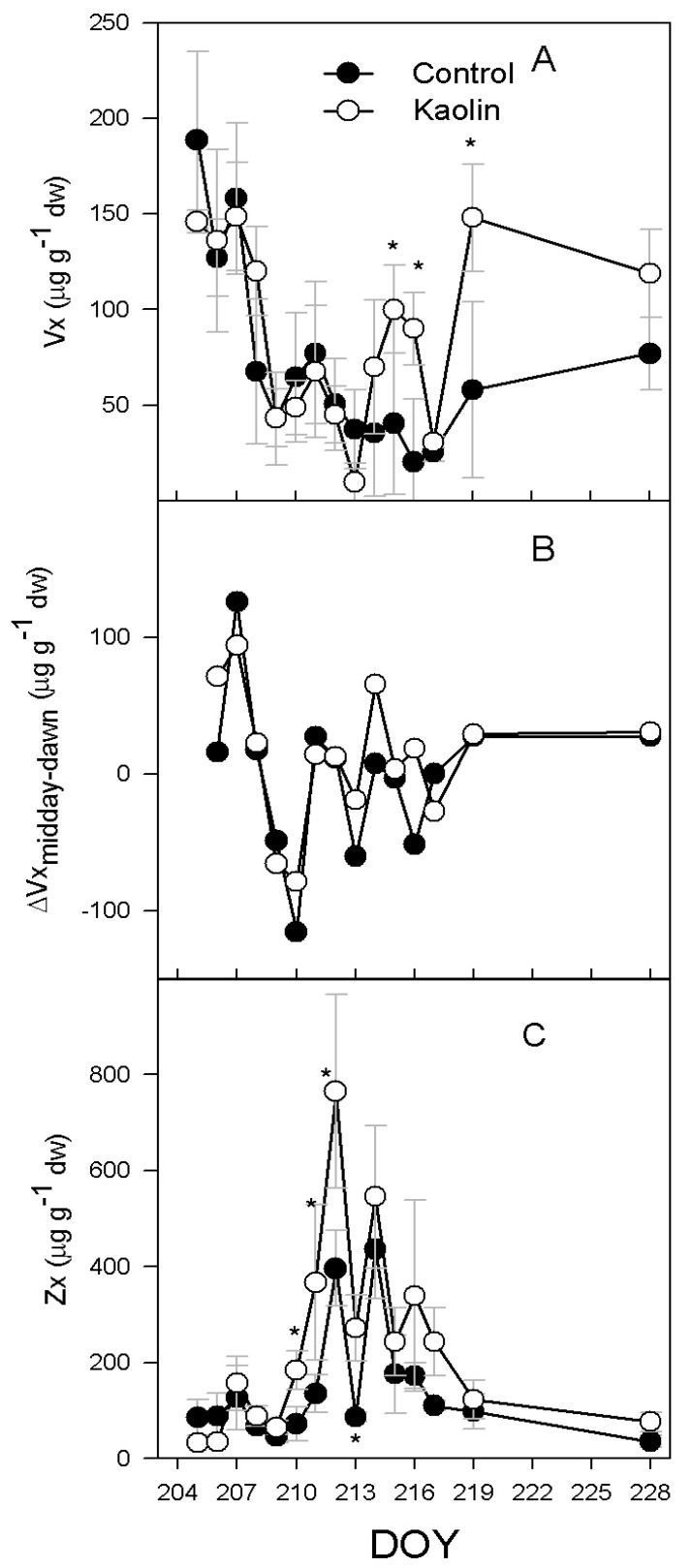
(**A**) Course of leaf violaxanthin (Vx) content at dawn; (**B**) midday to dawn Vx differences and (**C**) zeaxanthin (Zx) content at midday over the experiment, according to a progressive water shortage (DOY 209–217) and subsequent re-watering (at DOY 218), in vines subjected to the kaolin treatment and in controls. Bars represents standard error, *n* = 3. Asterisks indicate dates within which differences among treatment were significant (*p* < 0.05). DOY: day of the year.

Vx leaf concentration at 4:00 a.m. decreased in both treatments until DOY 213 (Figure 4A). From DOY 214 to DOY 216, kaolin vines had a higher leaf Vx concentration than control vines (53 µg g dw^−1^, if averaged over the three days). Although no difference was found at the peak of water deficit, higher leaf Vx concentration in kaolin vines was found again upon re-watering (90 µg g dw^−1^). The night recovery of Vx was variable during the experiment, yet no differences were found between treatments throughout the experiment (Figure 4B). From DOY 205 to DOY 209, kaolin did not affect leaf Zx concentration (Figure 4C). On DOY 210, Zx concentration was higher in kaolin leaves (184 ± 41 µg g dw^−1^ vs. 72 ± 36 µg g dw^−1^ found in control). Difference between treatments peaked on DOY 212 (369 µg g dw^−1^ in kaolin) and vanished once vines were re-watered.

**Figure 5:**
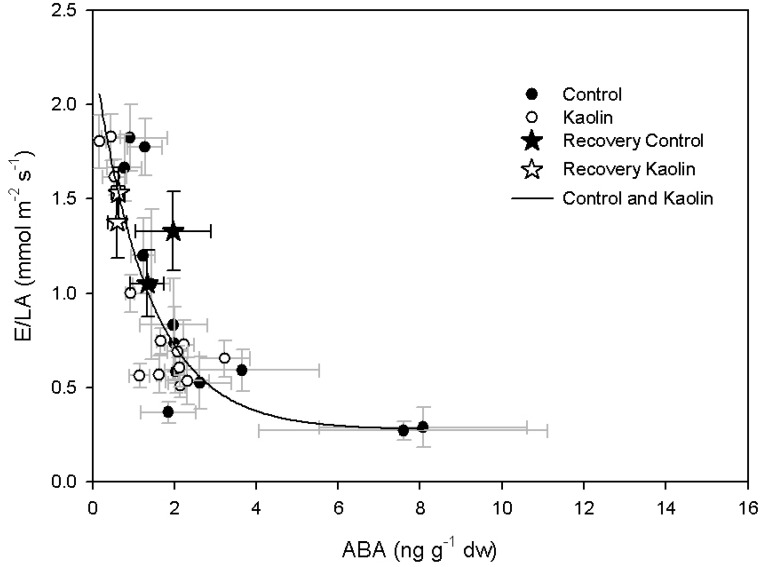
Correlation between whole-canopy transpiration rate/leaf area (E/LA) and leaf abscisic acid (ABA) content.

**Figure 6:**
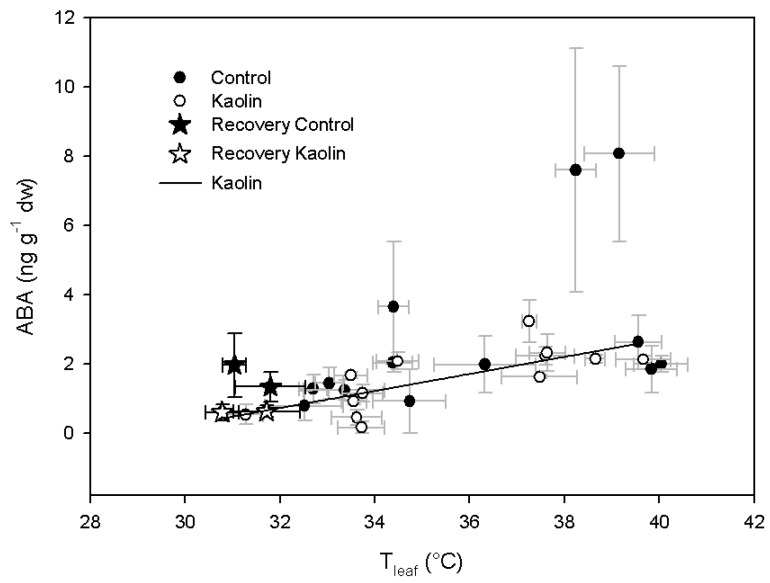
Correlation between leaf abscisic acid (ABA) content and leaf temperature (T_leaf_).

The correlation between E/LA and ABA was described by an exponential decay function (*y* = 0.28 + 2.02e^−0.75*x*^; *R*^2^ = 0.73, *p* < 0.0001), with no difference due to the treatments, although the range of ABA covered by kaolin vines was lower than those covered by control ones (Figure 5). In kaolin-treated vines, a significant positive linear correlation (*y* = −7.16 + 0.25*x*; *R*^2^ = 0.61, *p* < 0.005) was found for leaf ABA vs. T_leaf_ (Figure 6). On the contrary, in control vines leaf ABA was not correlated to varying T_leaf_.

## 3. Discussion

The regulation of transpiration occurs through the response of the whole vine physiology to multiple environmental signals and biochemical regulation. In this experiment, we limited the light absorbed by leaves by spraying kaolin, a natural clay inducing light reflection in the visible, infra-red and ultra-violet wavebands [8,30]. Kaolin-based formulates are widely used in many crops to reduce leaf and fruit sunburn damages [7,8,11,13,31,32,33]. In our experiment, kaolin was effective at increasing single leaf reflected light and at decreasing transmitted light (Table 1), in agreement with Rosati et al. [10] and Steiman et al. [30].

In our experiment, when vines were exposed to reduced water supply, Ψ_md_ decreased and leaf transpiration slowed down along with photosynthesis (Figure 1). Overall, the kaolin treatment had a moderate effect on transpiration rate, mainly exerted under severe water deficit, resulting in a reduction of T_leaf_. The difference of T_leaf_ between treatments appeared to be related to the larger transpiration, rather to the reduction of absorbed light; prior to water stress (WS) imposition, T_leaf_ was similar in the two treatments. On the other hand, during water shortage, T_leaf_ was significantly lower in kaolin in comparison with control.

Transpiration is regulated by stomata—stomatal conductance is influenced by environmental stimuli, such as light and leaf-to-air vapour pressure deficit, and by physiological regulation mechanisms that are usually divided into active, mainly ABA-mediated, and passive, mainly related to water potential [18,19,20]. In our experiment, we did not observe, between treatments, significant variation of stomata response to the decrease of leaf water potential and to the increase of leaf ABA (Figure 2 and Figure 5). The correlation between transpiration rate and leaf water potential was linear; instead, in the plot of E/LA vs. ABA, data were divided in three clusters as follows: data recorded before water deficit onset, when E/LA was above 1.5 mmol m^−2^ s^−1^; data recorded during reduced water supply; and the two points of the control vines recorded at the end of the water deficit period. During water recovery, E values were similar to those recorded during water stress. These data are consistent with previous studies that reported a decrease of transpiration as related to leaf water potential during the early stages of water shortage, followed by a significant increase of ABA after stomata closure [34,35]. However, this was not the case in kaolin vines, where ABA content did not significantly increase during the final stages of water deficit, as was instead the case in control vines. Overall, these data are in agreement with Dinis et al. [29], who reported a linear correlation between stomatal conductance (g_s_) and leaf ABA of field-grown grapevines, even though in their work kaolin-coated leaves had a looser response of g_s_ to leaf ABA concentrations, in comparison with control. Despite the evidence of a lower ABA content in kaolin-treated leaves, its cause is still unclear.

The ABA precursor is neoxanthin (Nx), which is formed from zeaxanthin (Zx), after the de-epoxidation of violaxanthin (Vx), via the intermediate antheraxanthin (Ax) formation. In our experiment, the amount of Vx + Ax + Zx, representing the molecules involved in the xanthophyll (VAZ) cycle, increased in the kaolin treatment, in comparison with control. Light plays an important role in the activation of the VAZ cycle—the regulation of violaxanthin de-epoxidase enzyme (VDE) is pH dependent and it is activated by the acidification of the thylakoid lumen when photosynthetic electron transport exceeds the capacity of assimilatory reactions [25]. In our experiment, the reduction of water supply promoted the de-epoxidation state of xanthophylls, while in control there was an increase of Nx and, in the kaolin treatment, the Nx content was almost constant during all the experiment (Figure 3C). This suggests that, theoretically, the kaolin treatment reduced the conversion of Zx into Nx by stimulating the activity of ZEP or by reducing the activity of neoxanthin synthase (NxS). ZEP is inhibited by light, and the epoxidation of Zx to Vx mainly occurs at night [26]. In our experiment, the increase of Vx after the night recovery (ΔVx_midday-dawn_) was similar (Figure 4B), although, in kaolin, the larger pool of Vx + Ax + Zx during the water stress period (Figure 3A) led to a significantly higher content, in comparison with control, at DOY 215 and 216 (Figure 4A). These data suggest a similar activity of ZEP in the two treatments. On the other hand, from DOY 210 to 213 and from DOY 216 to 217, there was a significantly larger content of Zx in kaolin, in comparison with control, whereas Nx was lower. Therefore, the conversion of Zx into Nx was likely limited in kaolin vines. Nx is the precursor of ABA, which followed the same dynamic of Nx in both treatments during the experiment, while in kaolin ABA had a modest increase during the experiment and, in control, ABA increased almost 4-fold. This could explain the further decrease of transpiration observed in control vines vs. kaolin ones from DOY 215 to 217. In kaolin, the lack of Nx increase limited the ABA leaf content when water deficit was more severe and leaf physiology more affected by leaf dehydration. Interestingly, the contrasting behaviour in the two treatments regarding Nx and ABA biosynthesis was not related to Ψ_md_, which was similar across the two treatments (Figure 1B), neither to T_leaf_, since ABA content was correlated to leaf temperature only in the kaolin treatment (Figure 6). Our results suggest that the higher ABA biosynthetic rates were primarily driven by the prolonged reduction of E, resulting in an increase of T_leaf_ concurrent to the increase of leaf ABA.

These results contribute to explaining the mechanisms involved in the kaolin-induced protection of canopy functionality, that is, in reflecting radiation and preserving thermoregulation, kaolin maintains a viable leaf VAZ cycle running. This, in turn, avoids the energy excess, potentially leading to damage to photosystems. An active VAZ cycle prevents the onset of ABA biosynthesis by reducing the accumulation of its precursors.

## 4. Materials and Methods

### 4.1. Plant Material and Treatment Layout

The experiment was carried out in 2018 in an outdoor area in Piacenza (44°55′ N, 9°44′ E), Italy, close to the Agriculture faculty, on 6 five-year-old cultivar Sangiovese (*Vitis vinifera* L.) vines (clone R10, grafted on SO4 rootstock) grown in 55l pots. The set of plants was similar to that used in Frioni et al. [15]. In this experiment, six vines were arranged along a single row having a 35° NE-SW orientation. Vines were cane pruned, trained to vertically shoot-positioned (VSP) trellis. Horizontal cane was 1 m long accommodating 9 nodes and it was raised 90 cm from the ground. The pots were filled with a mixture of loamy soil and peat (80:20 by volume, respectively) and kept well watered until the beginning of the water deficit. Pots were painted white before the trial started, to limit radiation-induced overheating. Each vine was fertilized twice (i.e., one week before and two weeks after bud-break) with 4 g of Greenplant 15 (N) + 5 (P_2_O_5_) + 25 (K_2_O) +2 (MgO) + micro (Green Has Italia, Cuneo, Italy). The six vines were then randomly divided into two treatments as follows: three vines were sprayed on 23 July (DOY 204) at 9:00 a.m. with a formulation of 100% aluminium silicate (Baïkal, Agrisynergie, Périgueux, France) diluted in water at 3% concentration (kaolin); the remaining three vines were assigned to the untreated control (control). The kaolin solution was carefully sprayed on both canopy sides with a shoulder pump.

For the dry-down setup, the same protocol described in Frioni et al. [15] was used. All the vines were kept well watered until DOY 208 (27 July, phenological stage BBCH77 according to Lorenz et al. [36]) by supplying a daily amount of 3600 ± 424 ml per vine, representing 110% actual canopy transpiration (E) concurrently measured by the whole-canopy system described hereafter over DOY 200–207. Irrigation was performed through an automated water supply system described by Poni et al. [37]. Starting on DOY 209 (28 July), a constant water deficit was imposed on all the vines by programming the water supply system to deliver daily to each vine only 70% of whole-canopy potential transpiration, calculated on the basis of the data collected prior to the water deficit imposition (DOY 200–207), until the achievement of severe water deficit conditions. Re-watering was performed on DOY 218 (6 August), restoring full water supply to all vines until dismantling of the chambers. During water shortage, each pot surface was covered with a plastic sheet to prevent infiltration of rain water and to minimize losses due to soil evaporation.

### 4.2. Whole-Canopy Gas Exchange

Whole-canopy net CO_2_ exchange rate/leaf area (NCER/LA) measurements were taken using the multi-chamber system reported in Poni et al. [37] with the configuration described in Frioni et al. [15]. To warrant unbiased comparison vs. canopy development, leaf area (LA) per vine was estimated as described in Gatti et al. [38] and NCER/LA (μmol CO^2^/m^2^s) computed accordingly. Since vines assigned to the two treatments had the same shoot number inside (~8) and outside (1) the chambers and, additionally, shoot growth along the cane was very uniform, measured E and NCER were estimated to be ~91% of total vine actual transpiration and assimilation.

The chambers were set up on each vine and continuously operated 24 h from DOY 200 (19 July, four days prior to kaolin sprays and eight days prior to the beginning of reduced water supply) until DOY 229 (17 August, 10 days after re-watering of WS plants). Daily data were screened to consider only data recorded between 11:00 a.m. and 3:00 p.m. (mean PAR > 1000 μmol photons m^−2^s^−1^) and in the absence of rain or unfavourable weather conditions. Ambient (inlet) air temperature was measured by shielded 1/0.2 mm diameter PFA-Teflon insulated type-T thermocouples (Omega Eng. INC, Stamford, CT, USA) and direct and diffuse radiation were measured with a BF2 sunshine sensor (Delta-T Devices, Ltd, Cambridge, UK) placed horizontally on top of a support stake next to the chambers enclosing the canopies. Ambient (inlet) relative humidity (RH) at each chamber’s outlet was measured by an HIH-4000 humidity sensor (Honeywell, Freeport, IL, USA) mounted upstream of the EGM-4 (PP system, Amesbury, MA, USA). Chambers were dismantled on 18 August (DOY 230).

### 4.3. Leaf Water Status, Temperature and Light Reflectance

Progression of water deficit was monitored by measuring midday stem water potential (Ψ_MD_). Ψ_MD_ was measured daily at 1:00 p.m. on days with clear sky on one leaf per vine. Measures were taken using a Scholander pressure chamber (3500 Model, Soilmosture Equip. Corp., Santa Barbara, CA, USA). Leaves were sampled from the shoots contained in the chamber using a custom-built zip-lock lateral access to the chamber.

On the same days, mean temperature (T_leaf_) was measured on a mature well-exposed leaf per vine inserted on the shoot kept outside of the chambers. On each date, one frontal thermal image per leaf was taken under full sunlight conditions at ~50 cm distance from the leaf itself, using the FLIR i60 infra-red thermal imaging camera (FLIR Systems Inc., Wilsonville, OR, USA). Thermal image analysis was carried out with the FLIR Tools software (FLIR Systems Inc).

On DOY 212 (31 July), the PAR transmitted through and reflected from the leaves was measured using a Sun System PAR-meter with external sensor (Sun System, Vancouver, WA, USA) as described by Taylor [39]. For the reflected PAR, the light sensor was downward oriented about 50 cm above a fully exposed leaf; for the transmitted PAR, the light sensor was upward oriented, holding it 50 cm below the leaf, on its projected shade. Measures were taken on DOY 212 (31 July) at 1:00 p.m. on two leaves per vine.

### 4.4. Xanthophylls and Abscisic Acid Determination

The leaf concentration of abscisic acid (ABA), violaxanthin (Vx), antheraxanthin (Ax), zeaxanthin (Zx) and neoxanthin (Nx) was determined on fully mature leaves sampled at 2:00 p.m. from the beginning to the last day of the experiment. Additionally, Vx was also analysed in leaves sampled at dawn (4:00 a.m.). Three leaves per treatment were cut, immediately wrapped in aluminium foil and dipped into liquid N_2_. In a preliminary experiment, we sampled 10 leaves and, after cutting them in two halves, we washed one half and we proceeded to Vx, Ax, Zx, Nx and ABA determination using the same procedure described below. No significant differences between washed and unwashed samples were found (*p* = 0.05).The samples were stored at −80 °C and then lyophilised. Lyophilised material was weighed (dry weight) and ground.

Vx, Ax, Zx and Nx extraction was carried out following the method by Yuan and Qian [40] with some modifications. First, 100 mg of lyophilised leaf were spiked with 100 µl of internal standard (100 mg/L β-apo-8’-carotenal). Extraction was carried out with 1 mL of ethyl acetate containing 0.1% BHT, kept in agitation for half an hour. After centrifugation at 1500 rpm for 5 min, the resulting upper layer was collected, whereas the lower layer was extracted again with 1 mL of ethyl acetate containing 0.1% BHT. The combined upper layer extracts were dried by evaporation at 30 °C under vacuum. The residue was resuspended in 1 mL of acetone containing 0.1% BHT and centrifuged at 11,000 rpm for 5 min. The clear extract was injected onto the HPLC. Each sample was extracted in triplicate. Sample handling, homogenization and extraction were carried out under reduced light and kept cold to minimize light-induced isomerization and oxidation of carotenoids. The concentration of carotenoids was determined on an Agilent 1260 Infinity HPLC (Santa Clara, CA, USA). A Luna 3 µm C8 (2) 100A, 100 × 4.6 mm column (Phenomenex, Castel Maggiore, Italy) was used with the setting temperature at 60 °C. The eluents were methanol and 1 M ammonium acetate (70:30) (solvent A) and 100% methanol (solvent B). Total flow rate was 1 mL/min. A binary gradient system was employed passing from 0 min (95% A 5% B) to 60 min (5% A; 95% B). Sample injection volume was 5 µL, and absorbance at 450 nm was used for quantification. β-Carotene was identified by comparison with retention time and UV spectra of commercial β-carotene standard (95% purity) (Sigma-Aldrich, St. Louis, MO, USA). Identification of the other carotenoids was performed by comparing retention time and UV–visible photodiode array spectra with authentic standards. Vx, Ax, Zx and Nx standards were purchased from CaroteNature (Münsingen, Switzerland). All the compounds were run in triplicate and calculated as β-carotene equivalent. De-epoxidation state was calculated as (Zx + 0.5Ax)/(Vx + Ax + Zx). Vx variation between midday and the next dawn (ΔVx_midday-dawn_) was calculated as the difference between the mean Vx content at midday and the mean Vx content at dawn per each treatment.

ABA was extracted following the procedure described by Vilarò et al. with some modifications [41]. Leaf material (0.1 g) was extracted with 10 mL of methanol/water (1:1 v/v, pH = 3 with formic acid) and homogenised for 3 min with an Ultra-Turrax homogenizer (IKA-Werke GmbH & Co., Staufen, Germany). After centrifugation (5000 rpm for 6 min), the supernatant was filtered through a paper filter and the same procedure was repeated for the remaining pellet. The collected filtrates were extracted twice with dichloromethane (15 ml) and the organic phase evaporated under vacuum. The residue was dissolved to a 100 µl acetone and 250 µL water/acetonitrile (70:30 v/v, 0.1% formic acid) for the HPLC analysis. Analytical standards of (±) abscisic acid (purity ≥ 98.5%) was purchased from Sigma-Aldrich; PA-grade methanol, acetone, dichloromethane and formic acid and HPLC-grade acetonitrile and water were purchased from VWR Chemicals (Milan, Italy). Analyses were performed with an Agilent 1260 Infinity HPLC (Santa Clara, CA, USA) equipped with a Sinergy 4 µm Hydro-RP (250 mm × 4.6 mm) column (Phenomenex, Castel Maggiore, Italy) with Security Guard at 35 °C, at a flow rate of 0.8 mL min^−1^; the injection volume was 20 μL and the detection was made at 265 nm. The mobile phase of acetonitrile/water (30:70 v/v, 0.1% formic acid) was previously filtered and degassed. The compound was identified by comparing the retention times with those of the authentic reference compound. The peaks were quantified by an external standard method, using the measurements of the peak areas and a calibration curve. Stock solutions of ABA standards were prepared by diluting a solution (10 mg mL^−1^ in acetonitrile) to obtain a range of concentrations from 0.01 to 10 mg mL^−1^. The limit of detection (LOD) was 0.005 mg L^−1^.

### 4.5. Statistical Analysis

Light reflection and transmission were analysed by a one-way analysis of variance (ANOVA), using Sigma Stat 3.5 (Systat Software, San Jose, CA, USA). Treatment comparison was performed by Student’s t-test at *p* ≤ 0.05. Data taken over time were analysed with the repeated measure analysis of variance routine embedded in the XLSTAT 2019.1 software package (Addinsoft, Paris, France). Least squares mean method at *p* < 0.05 was used for multiple comparisons within dates. Correlation between parameters was tested by regression analyses and all models were calculated using Sigma Plot 11.0 (Systat Software, San Jose, CA, USA). R^2^ significance was tested by ANOVA per *p* = 0.05.

## 5. Conclusions

Kaolin reduces the accumulation of ABA in the leaf by reducing the synthesis of Nx from Zx, resulting in faster recovery of vine gas exchange. These results further support the hypothesis that ABA mainly relates to its biosynthesis in leaves and that its accumulation can be limited by the downregulation of Nx synthesis. Moreover, this experiment provides the evidence that kaolin promotes the activity of the VAZ epoxidation/de-epoxidation cycle even under stressful conditions, thus preserving a full Vx pool after night recovery and a fluid energy dissipation of electron excess. Further experiments are needed to determine the biochemical pathway (gene expression and enzyme activity) leading to the downregulation of the Nx synthesis caused by kaolin.

## Figures and Tables

**Table 1:** Photosynthetically active radiation (PAR) reflected and transmitted at 1:00 p.m. by kaolin.

	Reflected PAR			Transmitted PAR		
	(% of Total PAR)			(% of Total PAR)		
Control	10.10%	±	0.88 b	8.30%	±	0.02 a
Kaolin	15.14%	±	0.45 a	6.86%	±	0.41 b

Different letters mean significant difference per *p* < 0.05 (t-test).
